# BRAZIL’S PROGRESS IN PROTECTING, PROMOTING AND SUPPORTING BREASTFEEDING FROM THE PERSPECTIVE OF THE GLOBAL BREASTFEEDING COLLECTIVE

**DOI:** 10.1590/1984-0462/2021/39/2019296

**Published:** 2020-08-28

**Authors:** Daiane Sousa Melo, Mariane Helen de Oliveira, Débora dos Santos Pereira

**Affiliations:** a Universidade de São Paulo, São Paulo, SP, Brazil.

**Keywords:** Breastfeeding, Public health policy, Infant nutrition, Aleitamento materno, Políticas públicas de saúde, Nutrição infantil

## Abstract

**Objective:**

To identify and to discuss the progress of actions for the protection, promotion and support of breastfeeding in Brazil from the perspective of the indicators proposed by the Global Breastfeeding Collective.

**Data source:**

A narrative review was conducted according to the methodological orientation of the implementation research and through a qualitative approach. Publications from the World Health Organization and the United Nations Children’s Fund were selected, as well as publications from the Brazilian Ministry of Health were collected from the Virtual Health Library and from the libraries of the Department of Primary Care’s portal and the Brazilian’s Institute of Geography and Research.

**Data synthesis:**

Brazil has shown promising results regarding the implementation of breastfeeding protection legislation, the participation of municipalities in community breastfeeding support programs, and the continued evaluation of these programs. However, reports of breastfeeding rates have not been produced every five years and the progress of these indicators is very far from the agreed targets for 2030. There is also a need to improve the number of births in child-friendly hospitals and financial donations for breastfeeding programs.

**Conclusions:**

It is necessary to strengthen systematic monitoring of breastfeeding and following up current strategies to more effectively impact the breastfeeding rates in the country. Furthermore, it is suggested that the practice of donations is a pathway to be explored to support breastfeeding programs.

## RESUMO

**Objetivo:**

Identificar e discutir o progresso das ações para a proteção e a promoção do aleitamento materno no Brasil, bem como o apoio a ele, sob a perspectiva dos indicadores propostos pelo *Global Breastfeeding Collective*.

**Fontes de dados:**

Foi realizada uma revisão narrativa de acordo com a orientação metodológica da pesquisa de implementação e por meio de uma abordagem qualitativa. Foram selecionadas publicações da Organização Mundial da Saúde e do Fundo das Nações Unidas para a Infância e também publicações do Ministério da Saúde do Brasil coletadas na Biblioteca Virtual em Saúde e nas bibliotecas do portal do Departamento da Atenção Básica e do Instituto Brasileiro de Geografia e Estatística.

**Síntese dos dados:**

O Brasil tem apresentado resultados promissores com relação à implementação da legislação para proteção do aleitamento materno e à participação dos municípios nos programas comunitários de apoio ao aleitamento materno e na avaliação continuada desses programas, contudo os relatórios das taxas de aleitamento materno não têm sido produzidos a cada cinco anos, e o progresso desses indicadores está muito distante das metas pactuadas para 2030. Também há necessidade de melhoria no número de nascimentos em hospitais amigo da criança e das doações financeiras para os programas em prol da amamentação.

**Conclusões:**

É necessário o fortalecimento do monitoramento sistemático do aleitamento materno e do acompanhamento das estratégias atuais para que impactem de maneira mais efetiva nas taxas de amamentação no país. Ademais, sugere-se que a prática de doações é um caminho a ser explorado para o apoio dos programas de amamentação.

## INTRODUCTION

On August 1, 2017, the Global Breastfeeding Collective (GBC) was launched, a partnership between 20 international agencies, including non-governmental and academic organizations, institutions and donors, led by the World Health Organization (WHO) and the United Nations Fund for Children (Unicef), aiming to universally improve investments in breastfeeding (BF). This initiative aims to go beyond the proposed goals agreed at the 71st World Health Assembly (WHA) and is in line with the 2030 Sustainable Development Goals timeline, in addition to adding explicit targets for other critical aspects of BF.^1^

Through scientific evidence on the potential of BF to save lives and promote a country’s socioeconomic development, many investments have been applied in global policies aimed at increasing BF rates and incentives. The GBC’s mission is to gather political, legal, financial and public support so that BF rates increase. Official publications of the cooperation proposal bring clear messages from the theoretical foundation to support investment in it:^1^

- BF is vital in ensuring health early in life. Breast milk is the best nutritional source, as it is complete with nutrients and unique immunomodulatory compounds, considered as a first vaccine that protects the baby’s health.
- BF can improve countries’ prosperity by decreasing the cost of treating preventable diseases and developing healthier people who will become a more skilled workforce.
- BF should not be only a woman’s job. The practice requires encouragement and support of qualified counselors and family members, as well as access to health services, support from employers, policy makers, and others.
- An investment of $ 570 million per year by governors, donors and partners over the next 10 years can help raise the prevalence of exclusive breastfeeding (EBF) by at least 50%. That amount would be equivalent to less than $ 5 per newborn to provide the support mothers need to breastfeed.

GBC calls countries to action with a view to providing mothers with the technical, financial, emotional and public support necessary to breastfeed in their baby’s first hour of life, breastfeed exclusively until 6 months of age, and continue BF with healthy complementary food, up to 2 years of age or more. Thus, seven political actions are proposed to implementers and donors from governments, philanthropic institutions, international organizations and civil society to achieve progress in the prevalence of BF and EBF:^2^

- **Financing:** increase investments in programs and policies that promote, protect and support BF.
- **International Code for the Marketing of Breast Milk Substitutes:** fully implement the code with legislation and effective application.
- **Protection of maternity in the workplace:** enact paid family leave policies and labor policies.
- **Baby Friendly Hospital Initiative (BFHI):** implement the “Ten Steps to Successful Breastfeeding” in maternity hospitals.
- **BF counseling and training:** improve access to BF specialist counseling in health facilities.
- **Community support programs:** encourage networks that protect, promote and support BF.
- **Monitoring systems:** track the progress of policies, programs and funding for BF.

The improvement in BF rates worldwide benefits the health and development conditions of children. Globally, every year, more than 820,000 children under the age of 5 could have their lives saved if the proportion of BF was increased.^3^

Since the baby’s first hour of life, BF is a potent protection for both maternal and child health, contributing to significantly reduce infant mortality and morbidity from respiratory infections and diarrhea, as well as the number of hospitalizations for these causes. Breastfeeding also promotes long-term benefits, such as lower risk of overweight in children and the prevention of 20,000 deaths per year from breast cancer in mothers, in addition to reducing the incidence of chronic non-communicable diseases (NCDs).^3^

Well-nourished children have better mental and cognitive development, a fact that favors reaching the global goals related to quality education, economic growth and job creation and, consequently, lower social inequality.^4^ In addition, a higher prevalence of BF may generate additional income for $ 302 billion annually, close to 0.5% of the world’s gross national income (GNI).^3^

Despite these important facts, less than half of the newborns have received BF in the first hour of life (42%) and EBF (41%) until 6 months of age, a scenario very far from the global goal for 2030, which would be to reach at least 70% in these indicators. Although approximately two-thirds of mothers continue to breastfeed throughout their child’s first year of life (71%), this rate drops to 45% at 2 years of age.^3^

In Brazil, many efforts have been made in this context since the 1970s, when the National Food and Nutrition Program (1976) and the National Breastfeeding Incentive Program (1981) were created. Since then, public health policies have had a positive impact on the practice of BF across the country, according to the results of national surveys over the past three decades. In the historical series of Brazilian BF indicators, there was an upward trend until 2006, with relative stabilization between 2006 and 2013, which was considered a sign of the need to evaluate and review policies and programs to promote, protect and support BF.^5^ ^,^ ^6^

Given this context, this study aimed to identify and discuss the level of progress of actions for the protection and promotion and support of BF in Brazil from the perspective of the indicators proposed by the GBC.

## METHOD

This study was conducted based on the methodological orientation of the implementation research, which focuses on the identification and description of the phenomena and their associations with the context in which the implementation of a given program takes place.^7^

The method of data collection selected was literature narrative review, which is useful to discuss the theory and contexts through the production of the synthesis of previous scientific publications on a certain topic. This methodology is indicated whenever the research question is not so specific as to be addressed with the systematic review design.^8^

Through a qualitative approach, a review of WHO and Unicef publications was carried out to obtain information about the GBC’s proposal and its methodology for analyzing countries’ progress and to identify the categorization of Brazil regarding the proposed indicators. Publications by the Ministry of Health were also reviewed, all containing data from the most recent national BF surveys and official publications on current national policies and programs for the protection, promotion and support of BF in the Virtual Health Library databases from the Ministry of Health (BVS MS), and in the libraries of the portal of the Department of Primary Care (Portal DAB) and the Brazilian Institute of Geography and Statistics (IBGE), so that it covered the most relevant documents in relation to the theme of this study.

## RESULTS AND DISCUSSION

With the objective of stimulating progress, increasing accountability and documenting changes in all countries, the GBC developed the countries’ Scorecard to monitor the progress of the seven political actions. This tool compiles global data on the status of policies and programs that affect BF rates, provides information on current BF rates and a scheme categorized into four levels by color (green, yellow, orange and red), in which green indicates the best level of progress and red the least advances.^9^ ^,^ ^10^

Each action has a progress indicator and a target to be met by 2030 that serve as a call for policy makers around the world to make BF support a collective national priority.

The Scorecard 2019’s indicators set the following parameters as the minimum target for actions to reach the green category:^10^

- At least US$ 5 in donations for each live birth.
- Full provision of the Code in legislation.
- Compliance with the provisions of Recommendation 191 (R191) (at least 18 weeks of maternity leave, 100% of previous earnings paid for a social program).
- ≥50% of births in BFHI-accredited hospitals and maternity hospitals.
- ≥75% of primary care services offering individual counseling for infants and young children (from the original, infant and young child feeding - IYCF).
- ≥75% of municipalities with community support programs for BF implemented.
- Assessment by the World Breastfeeding Trends Initiative (WBTi) since 2014.
- EBF data collected since 2014.

Although some countries have already made significant progress, no region has achieved green rating on all indicators yet.^2^ Furthermore, many of the standards set by these indicators are minimal recommendations. Thus, even countries that have favorable indicators should pay attention to carefully assess what can still be done to improve or maintain BF practices, so that they can guarantee a long-term sustainable goal.^10^

Globally, monitoring actions draw attention because they are the indicator that has been farthest from the target for 2030. GBC data show that, in 2018, 40% of countries had collected EBF data in the last five years and 43% of countries had evaluated BF programs in the same period.^3^

As for Brazil, the country has promising results in the implementation of the International Code of Marketing of Breast-Milk Substitutes, in the participation of municipalities in community support programs for BF, and in the continuous evaluation reports of these programs, as shown in Chart 1.

**Chart 1:** Levels of Brazil’s progress in the Global Breastfeeding Collective indicators, 2019.

Favorable environments and reports	Color code
$ 0.05 (USD) per child allocated for donor funding in 2013.	red
Legislation is fully enacted, or regulations, decrees or other legally binding measures are adopted covering all or almost all provisions of the Code and subsequent resolutions of the World Health Assembly.	green
The legislation provides for 17.2 weeks of maternity leave with 100% of previous earnings paid by social funds.	yellow
23.4% of births occurred in hospitals and maternity hospitals accredited by the Baby Friendly Hospital Initiative.	yellow
No data on percentage of primary health care services with individual counseling for feeding babies and young children.	grey
100% of the municipalities implement community nutrition, health or other programs with individual counseling for feeding babies and young children.	green
The most recent assessment by the World Breastfeeding Trends Initiative of the general breastfeeding program dates 2014.	green
Rates of exclusive breastfeeding were last measured in 2006.	orange

Source: United Nations Children’s Fund.^11^

The indicators with the lowest level of progress were the value of investments from donations and the monitoring of EBF rates in the country at least every five years. It is also worth highlighting the need to improve birth rates in hospitals and maternity hospitals accredited by BFHI, which do not reach half the minimum percentage desired for the green category. In the reference databases used by the Global Breastfeeding Scorecard, data on the percentage of primary care services that offer individual counseling on infant feeding in Brazil were not identified, as shown in Chart 1, in gray^11^.

Regarding the assessment of progress in monitoring EBF rates, the GBC used data available from the Unicef IYCF Database.^10^ The information about Brazil in this database refers to the numbers of the National Demographic and Health Survey of Children and Women (NDHS) of 2006.

More recent information on BF rates in Brazil was obtained by the National Health Survey (NHS) in 2013, however they are only available in IBGE microdata files, which can make it difficult for international organizations to access the most current reports.^6^ Still, in order to progress in this indicator, Brazil should have produced reports of EBF rates in 2018, but in the real scenario the next NHS is currently in the process of data collection, having started in the second half of 2019.^12^

To delineate a view of Brazil’s progress in the most recent BF indicators, Chart 2 shows data from the NDHS 2006 and the NHS 2013^13^ ^,^ ^14^. It is worth mentioning that, while the NDHS 2006 was designed to assess aspects related to maternal and child health, the focus of the NHS 2013 was the health situation of the adult population in general, without providing information on the rates of BF in the first hour of life.^6^

**Chart 2:** Levels of progress in breastfeeding rates in Brazil in relation to the Global Breastfeeding Collective 2019 indicators.

Indicator	Goal for 2030	NDHS 2006	Color code	NHS 2013	Color code
Breastfeeding in the first hour of life	>70%	42.9%	orange	--	
Exclusive breastfeeding in the first 6 months of life	≥70%	38.6%	orange	36.6%	orange
Breastfeeding in the first year of life	≥80%	47.5%	orange	45.4%	orange
Breastfeeding in the second year of life	≥60%	24.8%	orange	31.8%	orange

NDHS: National Demography and Health Survey of Children and Women; NHS: National Health Survey.

Source: United Nations Children’s Fund^10^, Brasil^13^ ^,^ ^6^ ^,^ ^14^.

One can see that only the indicator “BF at 2 years of age” made progress in the years between surveys. Therefore, there is much to improve when looking at the gap between the most recently monitored rates in BF and the targets set for 2030.

The main factors associated with the low prevalence of EBF in Brazil between 1998 and 2010 were low maternal education, low birth weight, and use of pacifiers. Access to information or guidance to mothers by professionals from primary health care services was directly associated with encouraging EBF practices; access to services such as the human milk bank (HMB) and hospitals accredited by BFHI was also considered a determining factor in this context.^15^

In view of the determinants of EBF practices in the country, one may suggest that investment in public programs and policies aimed at creating favorable environments and infrastructure that support women to breastfeed is essential for improving the current scenario in the country.

### Strengthening favorable environments for breastfeeding in Brazil

Many of the efforts undertaken through public health policies and programs have significantly impacted the practice of BF in Brazil since the 1970s. The National Food and Nutrition Program of 1976 and the National Health Program for Breastfeeding Incentive of 1981 can be considered as pillars of these proposals. The Brazilian Constitution of 1988 created the Unified Health System (SUS) and had its role of relevance to BF by establishing the right to maternity and paternity leave, and the right to women deprived of the freedom to remain with their baby during BF.^5^

Within the scope of SUS’s primary health care services, the “*Amamenta e Alimenta Brasil*” Strategy (“Breastfeed and feed” strategy, EAAB) currently plays an important role in the implementation of actions to protect and promote BF and healthy complementary food and support both across the country. EAAB is based on the critical-reflective methodology to improve the skills and work skills of the teams of professionals in Basic Health Units (BHUs). In the practical routine of the BHUs, trained tutors must support the planning and monitoring of activities to promote BF and healthy complementary food in at least one unit. Thus, a schedule of actions is agreed with the BHU team, in which tutors participate in monitoring and providing technical support.^16^

EAAB encourages compliance with the actions by providing BHU certification that meets the six proposed criteria. These criteria involve aspects similar to GBC’s progress indicators, including the development of systematic actions to promote BF, monitor BF indices, the requirement of a flow chart and protocol for the organization of child health care and compliance with legislation for protection of BF.^16^

The EAAB implementation process possibly helped the country to reach promising levels in the GBC’s progress indicators on the implementation of the code in the legislation, the participation of municipalities in community programs and the recent evaluation reports of BF promotion programs, as mentioned previously in Chart 1. The overall assessment of the strategy’s implementation, carried out in 2018, showed that EAAB was successful in creating permanent education actions: since the beginning of the implementation, in 2013, 40,246 primary health care professionals were qualified. On the other hand, the data also show that just over 100 BHUs were certified and that the active participation of trained tutors has weakened, presenting a low level of monitoring of the actions.^17^

This scenario indicates that, despite the advances, there are critical points in the EAAB implementation process that should be investigated so that such investment in the qualification of professionals is in fact converted into results of greater impact in the creation of favorable environments to increase the rates of BF and healthy complementary food in the first 2 years of children’s lives.

It is also important to consider that flawed aspects in the actions to monitor the implementation of the strategy may be related to the lack of reports on the coverage of actions supporting BF and on individual counseling, a fact similarly observed in the Global Breastfeeding Scorecard data, which identified lack of information from countries on the number of women covered by community programs and on the quality of services provided.^2^ ^,^ ^17^

In the context of secondary care services, BFHI has played a valuable role in mobilizing hospital staff to change behaviors and routines regarding the high rates of early weaning. The process for designating an establishment as a Baby-Friendly Hospital begins with the hospital’s self-assessment, followed by external assessments of the criteria reached in relation to the restriction of breastmilk substitutes and the implementation of the ten steps for the success of BF.^18^

Researchers show that, in Brazil, children born in hospitals accredited by the initiative are more predisposed to receiving BF in the first hour of life (9%) and have significantly less use of pacifiers. Furthermore, the practice of EBF is 13% more common among children under the age of 2 months, as well as among those aged 3 (8%) and 6 months (6%).^19^

Studies claim that, despite BFHI playing a significant role in increasing rates of BF and EBF, many institutions face challenges to fully comply with the 10 proposed steps, mainly in aspects associated with the training of the entire health care team in the practices required to implement this policy.^20^

Considering that in Brazil only 23.4% of births occur in Baby-Friendly Hospitals, experts suggest that to increase the impact of hospitals and maternity hospitals, it would be necessary to create a Breastfeeding Committee at the beginning of the accreditation process, which would be responsible for coordinating habilitation activities and organizing the training of professionals.^20^

Another aspect to be taken into account about BFHI is that its positive impact on EBF rates decreases over time, which reinforces the need for continuous support in primary care services to encourage women to practice EBF until the sixth month of life of their babies.^19^

In addition to the important role of the contribution of federal initiatives to create favorable environments for BF through public policies, the GBC also emphasizes the relevance of financial donations by non-governmental institutions to BF programs and their significant correlation with the improvement of BF rates.^2^

The value of the GBC financial donor indicator was calculated by dividing the total value of financial donations to EBF by the number of live births in a country, based on data from Invest in Nutrition.^10^ Only 6% of countries receiving international aid earn at least five dollars per birth to support BF programs. Most countries receive less than one dollar per birth.^2^ In this study, it was clear that, in Brazil, the practice of donations to invest in EBF is very weak in relation to international expectations, which indicates a way to support BF that can be more explored in the country.

## FINAL CONSIDERATIONS

The literature narrative review made it possible to identify that, in recent decades, strong investment has been applied to protect, promote and support quality BF in Brazil through public policies and health programs. The main limitation of this review was the selection bias, since a narrative review adopts the subjectivity of the authors when evaluating and selecting articles; however, it is considered that the limitation was minimized by the quality of the publications selected for the study.

Under the analysis of the GBC indicators, the country has shown favorable results in the implementation of legislation to protect BF, in the participation of municipalities in community support programs for BF, and in the continuous evaluation of these programs.

Nevertheless, the review also brought evidence that the monitoring of EBF rates in the country has not occurred at least every five years and that BF rates in the first two years of life are very far from the targets agreed for 2030. Also, there is a need to increase the number of hospitals and maternity hospitals accredited by the BFHI, and the practice of financial donations for BF programs must be more widespread in the country.

Therefore, it is necessary to strengthen the systematic monitoring of BF practices and better integration with international databases. Also of great importance is the evaluation and monitoring of strategies currently used so that they impact more effectively and accelerate the pace of growth in BF rates across the country. Finally, the practice of financial donations for programs in favor of AM suggests a path that can be explored for the benefit of children’s, families’ and societies’ health.
